# Cell-Free Protein Synthesis with Fungal Lysates for the Rapid Production of Unspecific Peroxygenases

**DOI:** 10.3390/antiox11020284

**Published:** 2022-01-30

**Authors:** Marina Schramm, Stephanie Friedrich, Kai-Uwe Schmidtke, Jan Kiebist, Paul Panzer, Harald Kellner, René Ullrich, Martin Hofrichter, Katrin Scheibner

**Affiliations:** 1 Institute of Biotechnology, Brandenburg University of Technology Cottbus-Senftenberg, Universitätsplatz 1, 01968 Senftenberg, Germany; stephanie.friedrich@izi-bb.fraunhofer.de (S.F.); kai-uwe.schmidtke@b-tu.de (K.-U.S.); jan.kiebist@izi-bb.fraunhofer.de (J.K.); paul.panzer@b-tu.de (P.P.); katrin.scheibner@b-tu.de (K.S.); 2 Fraunhofer Institute for Cell Therapy and Immunology, Branch Bioanalytics and Bioprocesses, Am Mühlenberg 13, 14476 Potsdam-Golm, Germany; 3 Department of Bio- and Environmental Sciences, TU Dresden-International Institute Zittau, Markt 23, 02763 Zittau, Germany; harald.kellner@tu-dresden.de (H.K.); rene.ullrich@tu-dresden.de (R.U.); martin.hofrichter@tu-dresden.de (M.H.)

**Keywords:** unspecific peroxygenase, EC 1.11.2.1, monooxygenase, cell-free protein synthesis, in vitro translation

## Abstract

Unspecific peroxygenases (UPOs, EC 1.11.2.1) are fungal biocatalysts that have attracted considerable interest for application in chemical syntheses due to their ability to selectively incorporate peroxide-oxygen into non-activated hydrocarbons. However, the number of available and characterized UPOs is limited, as it is difficult to produce these enzymes in homologous or hetero-logous expression systems. In the present study, we introduce a third approach for the expression of UPOs: cell-free protein synthesis using lysates from filamentous fungi. Biomass of *Neurospora crassa* and *Aspergillus niger*, respectively, was lysed by French press and tested for translational activity with a luciferase reporter enzyme. The *upo1* gene from *Cyclocybe (Agrocybe) aegerita* (encoding the main peroxygenase, *Aae*UPO) was cell-free expressed with both lysates, reaching activities of up to 105 U L^−1^ within 24 h (measured with veratryl alcohol as substrate). The cell-free expressed enzyme (cf*Aae*UPO) was successfully tested in a substrate screening that included prototypical UPO substrates, as well as several pharmaceuticals. The determined activities and catalytic performance were comparable to that of the wild-type enzyme (wt*Aae*UPO). The results presented here suggest that cell-free expression could become a valuable tool to gain easier access to the immense pool of putative UPO genes and to expand the spectrum of these sought-after biocatalysts.

## 1. Introduction

Over the last two decades, biocatalysis has increasingly developed into a mature and widely applicable technology for chemical synthesis and production [1]. A number of bio-catalytic approaches have been developed for the environmentally friendly and effective synthesis of a range of chiral products, such as pharmaceuticals, agrochemicals, and fine chemicals. However, further development of these biocatalytic processes on an industrial scale will depend crucially on the widespread availability of efficient and robust enzymes [2,3].

Unspecific peroxygenases (EC 1.11.2.1) are enzymes found in numerous fungi and are ideally suited for the synthesis of specialty chemicals. As extracellular enzymes, they are inherently stable and self-sufficient, as evidenced by their ease of activation by hydrogen peroxide and independence of electron transport proteins, electron donors, or cofactors other than heme-stabilizing magnesium ions [4,5]. They have become ‘dream catalysts’ in organic synthesis because of their ability to selectively incorporate peroxidic oxy-gen into non-activated hydrocarbons, causing hydroxylations and epoxidations that are difficult to achieve by chemical methods [6,7,8,9]. Essential for this type of reaction is the heme cofactor that is coordinated proximally by the thiol of a cysteine residue. Due to their P450-like catalytic mechanism and reaction spectrum, UPOs can be used for the conversion of pharmaceuticals and drugs, e.g., of volixibat (*N*-dealkylation) [10], cyclophosphamide (aliphatic hydroxylation) [11], propranolol (aromatic hydroxylation) [12], testosterone (epoxidation) [13], corticosteroids (side-chain removal by C-C scission) [14] and clopidogrel (sulfoxidation and epoxidation) [15].

A crucial drawback in the application of UPOs (representing a diverse superfamily of proteins) and the main reason why their scope is still very narrow is their low avail-ability, limited to a few representatives [16]. Contrary to their undemanding nature in catalysis, UPOs are demanding when it comes to their expression. To date, there are only a few reports demonstrating the efficient production and purification of UPOs using wild-type fungi [13,14,17,18,19,20]. To circumvent the disadvantages of homologous production, such as low production rates, long production times, or high concentrations of contaminating proteins, various attempts have been made to express UPOs heterologously in established hosts. An overview of recombinantly expressed UPOs is given in the recent review of Kinner and colleagues [21]. Another peroxygenase from *Hypoxylon* that has been recently produced in *Pichia pastoris* (syn. *Komagataella phaffii*) can be added to this list [22].

However, even when using quite different host organisms, such as *Saccharomyces cerevisiae*, *P. pastoris*, *Aspergillus* spp., or *Escherichia coli*, it has not yet been possible to establish a universal expression system for UPOs. Overall, the number of available UPOs today is less than 20, which is in stark contrast to the number of putative UPO genes in fungal genomes, which is in the range of several thousand [20]. To close the gap between theoretically and practically available UPOs and to gain access to new catalytically promising candidates, a third option (besides homologous and heterologous expression) was chosen in this study: the production of UPOs by cell-free protein synthesis (CFPS).

There are examples of functional heme-containing enzymes that were cell-free expressed using lysates from *E. coli*: manganese peroxidase [23,24], a P450 monooxygenase [25], cytochrome c oxidase [26], and horseradish peroxidase [27]. This demonstrates the general potential of CFPS for the production of enzymes with such a complex prosthetic group; however, similar results have not yet been published regarding UPOs.

In general, CFPS can be considered a powerful and versatile tool for the rapid discovery of new biocatalyst candidates or improved biocatalyst variants [28]. Optimized systems are also of interest for the preparative production of proteins with yields in the milligram-per-milliliter range. There are CFPS systems based on bacterial, plant, archaeal, fungal, and animal cells. The most relevant and well-studied lysates used for CFPS are from *E. coli*, *Spodoptera frugiperda* (insect), *S. cerevisiae* (yeast), Chinese hamster ovary (CHO), rabbit reticulocyte, wheat germ, and HeLa cells [29]. To date, filamentous fungi just play a minor role in CFPS. In some published work, lysates from *Neurospora crassa* have been employed to evaluate the potential for the production of cell-free expressed proteins using reporter proteins or for in vitro studies as such, for example, to investigate the impact of codon usage [30,31,32,33]. Regarding the genus *Aspergillus*, there is only one publication dealing with the use of a lysate from *A. nidulans* in CFPS [34]. No studies have been published on *A. niger* in the context of CFPS.

*N. crassa* and *A. niger* are filamentous fungi from the phylum Ascomycota, but belong to different classes (Sordariomycetes and Eurotiomycetes, respectively). Based on bioinformatic analyses, both organisms carry putative UPO genes in their genomes [20]. There are good reasons for using filamentous fungi in CFPS, such as their innate competence for post-translational modifications, their ability to secrete proteins, and also their low requirements for the growth medium, which makes the preparation of lysates inexpensive.

In this article, we report the preparation of a functional UPO by a novel cell-free synthesis approach using lysates from *N. crassa* and *A. niger*. The cell-free UPO was tested in a substrate screening that included typical UPO substrates as well as some pharmaceuticals, and its biocatalytic activity was compared with that of its homologous counterpart (wild-type UPO).

## 2. Materials and Methods

### 2.1. Lysate Preparation

#### 2.1.1. Cultivation

*Neurospora crassa* (DSM 1257) was cultured in 500-mL Erlenmeyer flasks, each containing 200 mL of HA complete medium (10 g L^−1^ glucose and 10 g L^−1^ yeast extract) [35]. Each flask was inoculated with one drop of spore suspension (~10^7^ spores mL^−1^ in glycerol) and incubated for 48 h on a rotary shaker (Multitron 2, Infors AG, Bottmingen, Switzerland) at 34 °C and 120 rpm.

*Aspergillus niger* (DSM 11167) was cultivated in 500-mL Erlenmeyer flasks, each containing 100 mL of 2HA-MS medium [36], but without antifoaming agent. After inoculation with one drop each of spore suspension (~3 × 10^8^ spores mL^−1^ in glycerol), the flasks were incubated on a rotary shaker (Multitron 2, Infors AG, Bottmingen, Switzerland) at 30 °C and 150 rpm for 24 h.

#### 2.1.2. Preparation of Cell Lysates

Mycelium was harvested by vacuum filtration and washed twice with 50 mL of 4 °C cold mannitol buffer A [37]. Hereafter, the mycelium was resuspended in 1.5 mL of cold lysis buffer A [37] per g fresh weight. Cell disruption was performed by French press (HTU Digi-F-Press, G. Heinemann Ultraschall- und Labortechnik, Schwäbisch Gmünd, Germany) at 4 °C and 5000 psi (*N. crassa*) and 10,000 psi (*A. niger*), respectively. The raw lysate was centrifuged at 6500× *g* and 4 °C for 5 min. The supernatant was placed in a clean tube and clarified again under the same conditions; then the top 90% of the supernatant was collected without cell debris. All steps were carried out as quickly as possible and the samples were kept on ice in between. Lysis efficiency was monitored microscopi-cally.

To remove endogenous mRNA, micrococcal nuclease was added to the supernatant and incubated for 10 min at room temperature [37]. If not used immediately, aliquots (100–500 µL) of the lysate were snap-frozen in liquid nitrogen and stored at −80 °C.

### 2.2. Cell-Free Protein Synthesis

#### 2.2.1. Preparation of mRNA Templates

The synthetic gene of *Cyclocybe (Agrocybe) aegerita* UPO (*Aae*UPO; UniProtKB/Swiss-Prot: B9W4V6) was purchased from Invitrogen (Thermo Fisher Scientific) with a codon optimized sequence for *S. cerevisiae* containing a C-terminal six histidine peptide, cloned into a pYES2 vector. For run-off transcription, the plasmid was linearized with BstEII-HF (New England Biolabs GmbH, Frankfurt am Main, Germany) and column purified by using the Monarch PCR & DNA cleanup kit (New England Biolabs GmbH, Frankfurt am Main, Germany). In vitro transcription was done with the HiScribe™ T7 ARCA mRNA Kit (with tailing) (New England Biolabs GmbH, Frankfurt am Main, Germany) using 1 µg of the linearized plasmid as template to get mRNA with 5′-Cap and poly(A) tail. Synthesized mRNA was column purified using the Monarch RNA cleanup kit from New England Biolabs GmbH (Frankfurt am Main, Germany), checked for quality by gel electrophoresis, and quantified photometrically at 260 nm.

#### 2.2.2. Cell-Free Protein Synthesis

For the in vitro translation, a translation mix was freshly prepared containing 80 mM HEPES-KOH pH 7.4, 4 mM ATP, 0.4 mM GTP, 8 mM dithiothreitol, 80 mM creatine phosphate, 200 mM potassium acetate, 4.8 mM magnesium acetate, 0.24 mg mL^−1^ tRNA from yeast, amino acid mix (0.08 mM each) and 0.24 U µL^−1^ creatine phosphokinase. The cell-free reaction mixture consisted of 25% translation mix, mRNA (typically 28 nM) in DEPC water, and 50% fungal lysate. No-template controls contained only DEPC water instead of mRNA. Typical reactions were carried out in a volume of 20 µL in 1.5-mL reaction tubes (Eppendorf, Germany) in an incubator at 18 °C. In case of the reporter firefly luciferase, 28 nM mRNA (purchased from Promega, Walldorf, Germany) were used for CFPS reactions.

### 2.3. Analytics

#### 2.3.1. Enzymatic Assays

For measuring the activity of the reporter firefly luciferase, the ONE-Glo™ Luciferase Assay System (Promega, Walldorf, Germany) was used. 10 µL of the CFPS reaction were added to 40 µL of Luciferase Assay Buffer containing Luciferase Assay Reagent in a white 96-well plate. Prior to measurement, the plate was shaken for 60 s at 300 rpm in the plate reader. Light emission resulting from the luciferase-catalyzed oxidation of the substrate luciferin was detected with a CLARIOstar Plus plate reader (BMG Labtech, Ortenberg, Germany).

The activity of functional cell-free UPO was routinely tested with an LC-MS-based propranolol assay. The assay reaction (100 µL total volume) was initiated by adding 90 µL of a reaction mixture (1 mM propranolol, 1 mM hydrogen peroxide, 5 mM ascorbate, 20 mM potassium phosphate buffer, pH 7.0) to 10 µL of cell-free reaction mixture. The reactions were incubated at 25 °C and 800 rpm on a thermal shaker (TurboShaker 3500, Scienova GmbH, Jena, Germany) for 5 min and stopped by adding 100 μL of ice-cold ace-tonitrile (−20 °C). Subsequently, the samples were centrifuged at 14,000× *g* for 10 min, the supernatants were transferred to HPLC vials and analyzed by liquid chromatography-mass spectrometry (LC-MS). The UPO activity is given in propranolol units, where one unit (1 U) corresponds to the oxygenation of 1 μmol of propranolol to 5-hydroxypropra-nolol (5-OHP) in 1 min. In order to quantify the formation of 5-OHP, 4-hydroxypropra-nolol (Sigma Aldrich, Schnelldorf, Germany) was used for the calibration (as internal standard).

For the comparative study of UPO activities, we measured the conversion of the typi-cal UPO substrates veratryl alcohol, 2,2′-azino-*bis*(3-ethylbenzothiazoline-6-sulfonic acid) diammonium salt (ABTS), 5-nitrobenzodioxole (NBD), and naphthalene as well as of the pharmaceutical substrates propranolol, diclofenac, and clopidogrel. The CFPS reaction mixtures were sonicated (3 × 10 s pulses with 30 s pauses in between; amplitude 10%; Sonopuls, Bandelin, Berlin, Germany) to release the enzyme from the microsomes. The lysed samples were centrifuged at 16,000× *g* and 4 °C for 10 min and the supernatants were used for the reactions. Homologous *Aae*UPO (wt*Aae*UPO) was produced and purified as described elsewhere [17]. Both enzyme preparations were diluted to 100 U L^−1^ (based on the oxidation of veratryl alcohol to veratraldehyde; ε_310_ = 9300 M^−1^cm^−1^). The formation of the ABTS cation radical (ε_420_ = 36,000 M^−1^ cm^−1^), 5-nitrocatechol (ε_425_ = 9700 M^−1^ cm^−1^) and 1-naphthtol (ε_303_ = 9700 M^−1^ cm^−1^) were measured photometrically (CLARIOstar Plus, BMG Labtech, Ortenberg, Germany). The comparative conversion of the pharmaceutical substrates was followed analogously to the propranolol assay, and the products were quantified by LC-MS as described below. Additional calibrations were performed with the authentic standards 4′-hydroxydiclofenac and 2-oxoclopidogrel.

#### 2.3.2. High-Resolution Mass Spectrometry 

Chromatographic separations in LC-MS experiments were performed on a Thermo Scientific Vanquish Flex Quaternary UHPLC system (Thermo Fisher Scientific, Waltham, MA, USA) using a Kinetex^®^ column (C18, 2.6 µm, 100 Å, 150 × 2.1 mm, Phenomenex, Torrance, CA, USA). The injection volume was 1 µL, and the column was eluted at a flow rate of 0.5 mL min^−1^ and 40 °C with two mobile phases: A (diH_2_O, 0.1% formic acid) and B (acetonitrile, 0.1% formic acid) and following gradient: 0 min 10% B; 0.5 min, 10% B; 5 min, 80% B; 7 min, 80% B; 7.1 min, 10% B; 10 min, 10% B.

MS spectra were obtained using the Thermo Scientific Q Exactive Focus quadrupole-Orbitrap mass spectrometer (Thermo Electron, Waltham, MA, USA) coupled with a heated electrospray ionization source in positive mode. The tune operating parameters were as follows: rate of sheath gas flow and auxiliary gas flow—40 and 15 (arbitrary units), respectively; spray voltage—4.0 kV; temperature of capillary and sample heater—260 °C and 400 °C, respectively; high-resolution MS was operated at full scan positive mode with a mass range of m/z 150–1500 at a resolution of 70,000 (m/z 200).

#### 2.3.3. Western Blot

If needed, the CFPS samples were fractionated by centrifugation at 16,000× *g* and 4 °C for 10 min prior to Western blot analyses. The supernatant was transferred to a new 1.5-mL reaction tube, and the pellet was resuspended in 20 mM HEPES buffer pH 7.0 with the same volume as the supernatant. 

Protein samples (10 µL) were mixed with Laemmli sample buffer, heated at 95 °C for 5 min, and separated on Bolt 10% SDS-PAGE by using a mini gel tank (Life Technologies, Carlsbad, CA, USA). Subsequently, the proteins were transferred to a polyvinyl difluoride membrane with 0.2 µm pore size (Carl Roth, Karlsruhe, Germany). After blocking the membranes with 5% BSA in PBST with 0.3% Tween 20 for 1 h, the membranes were incubated with a monoclonal 6X-His epitope tag antibody (MA1-135, clone 4E3D10H2/E3, Life Technologies, Carlsbad, USA) at a dilution of 1:500 overnight at 4 °C, washed in PBST with 0.3% Tween 20, and probed with an HRP-conjugated anti-mouse IgG secondary antibody at a dilution of 1:1000 (#7076, Cell Signaling Technology, Danvers, MA, USA) for 1 h at room temperature. A light-emitting chemiluminescent substrate (SignalFire ECL Reagent, Cell Signaling Technology, Danvers, MA, USA) was used for the detection by exposing it with the biostep Celvin S chemiluminescence imager (biostep, Burkhardtsdorf, Germany). Densitometric evaluation of the protein bands was done by means of the software ImageJ (https://imagej.nih.gov/ij/; accessed on 9 December 2021; version 1.53k).

#### 2.3.4. Deglycosylation

CFPS samples (either fractionated as described above or as total reaction mix) were deglycosylated using the Protein Deglycosylation Mix II (New England Biolabs GmbH, Frankfurt am Main, Germany). The samples were subsequently analyzed by Western blot.

## 3. Results and Discussion

### 3.1. Lysate Preparation from Neurospora crassa and Aspergillus niger

**Figure 1:**
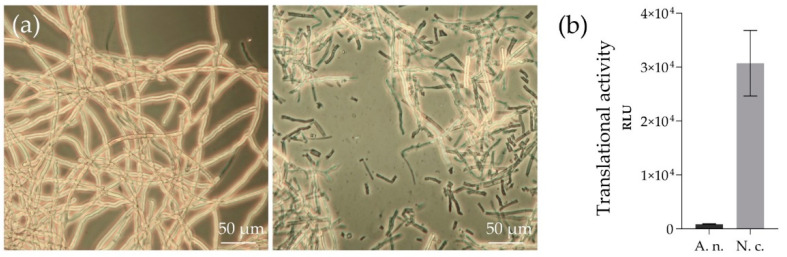
Lysis of fungal cells and testing of translationally active lysates from *N. crassa* and *A. niger*. (**a**) Mycelium of *N. crassa* prior to (left panel) and after (right panel) lysis with a French press homogenizer. Intact cells appear bright. (**b**) Cell-free expressed luciferase with lysates from *A. niger* (A. n.) and *N. crassa* (N. c.). The 20-µL reactions were incubated at 18 °C for 90 min and tested for luciferase activity by measuring the oxidation of luciferin via luminescence detection. The experiment was done in duplicate. RLU—relative luminescence units.

*N. crassa* and *A. niger* were grown in nutrient-rich media for 48 h and 24 h, respectively. Subsequently, the mycelia were harvested by vacuum filtration and yielded fresh weights of 25 to 30 g L^−1^ for both fungi. For cell disruption, a French press homogenizer was used, which represents a simple and fast method and is routinely used in the preparation of lysates from other fungal organisms such as *S. cerevisiae* [38]. The lysis was successful as 80 to 90% of the cells were broken, which was microscopically detectable (Figure 1a).

The lysates were tested for translational activities by using the cytosolic reporter enzyme ‘firefly luciferase’ (Figure 1b). With both lysates, significant amounts of firefly luci-ferase were in vitro translated, albeit in differing quantities. The measured values for *N. crassa* (30,721 ± 6091 RLU) were 35-times higher than those of *A. niger* (862 ± 78 RLU).

### 3.2. Cell-Free Protein Synthesis of AaeUPO

For our study on the cell-free production of a UPO, we used the *upo1* gene from *Cyclocybe (Agrocybe) aegerita*, which encodes the first reported UPO [17]. The extracellular heme-thiolate enzyme contains an N-terminal signal peptide followed by a propeptide and has a C-terminal intramolecular disulfide bond as well as six potential *N*-glycosylation sites [39]. For easier detection, the UPO gene was tagged with a C-terminal polyhistidine peptide. To reduce the complexity of the CFPS system, the mRNA of *upo1* was synthesized independently from the translation system and provided externally. The CFPS reactions were tested for the formation of functional cell-free UPO with an LC-MS-based propranolol assay since the formation of 5-hydroxypropranolol (5-OHP) from propranolol is characteristic of *Aae*UPO [12,40]. By using this assay, we could exclude the possibility of measuring false positive activities from potentially interfering endogenous monooxygenases (cytochrome P450 enzymes). Nevertheless, negative controls that contained no mRNA templates were additionally included in all experimental setups.

**Figure 2:**
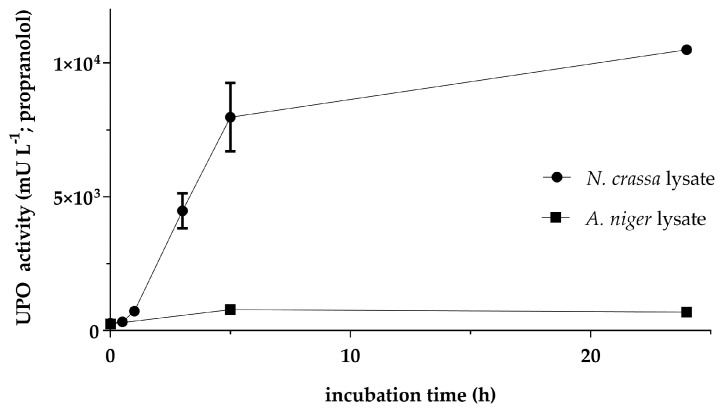
Time-dependent activity of cell-free synthesized *Aae*UPO with lysates from *N. crassa* and *A. niger*. The 20-µL reactions were prepared in separate tubes, incubated at 18 °C for the indicated time, and tested for UPO activity by measuring the formation of 5-OHP from propranolol by LC-MS analysis. Values are means ± standard deviation.

**Figure A1:**
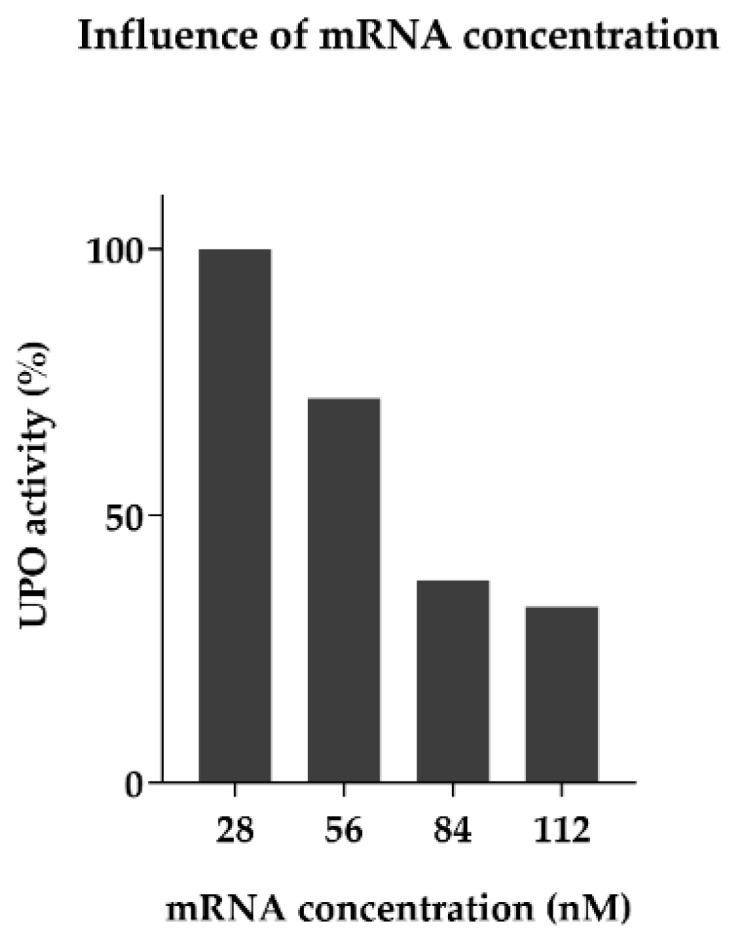
Cell-free synthesis of *Aae*UPO protein with lysate from *N. crassa* using varying concentrations of mRNA. 20-µL reactions with varying mRNA concentrations were incubated at 18 °C for 4 h and tested for UPO activity by measuring the conversion of propranolol to 5-OHP by LC-MS. The highest value (at 28 nM mRNA) was set to 100%.

Cell-free production of *Aae*UPO was evaluated with lysates from *N. crassa* and *A. niger*, respectively. Essentially, the reactions were set up according to Wu et al. with minor modifications [33]. The mRNA carrying the coding sequence for the UPO was applied at 28 nM since higher concentrations had adverse effects on the synthesis of active enzyme (Figure A1). The formation of active cf*Aae*UPO was observed over a period of 24 h (Figure 2). With both lysates, significant UPO activity could be detected: 10,498 ± 28 mU L^−1^ using the *N. crassa* lysate and 783 ± 18 mU L^−1^ with the *A. niger* lysate. The different yields of active enzyme correlated with the results for the reporter luciferase (Figure 1b). Compared to the lysate from *N. crassa*, the yield of active enzyme with the *A. niger* lysate was 10-times (after 5 h incubation) to 15-times (after 24 h incubation) lower. Interestingly, in contrast to *N. crassa*, the UPO activity with the *A. niger* lysate was higher after 5 h of incubation than after 24 h. This decrease in activity could indicate residual proteolytic activity in the lysate, despite the application of protease inhibitors during the preparation process.

**Figure 3:**
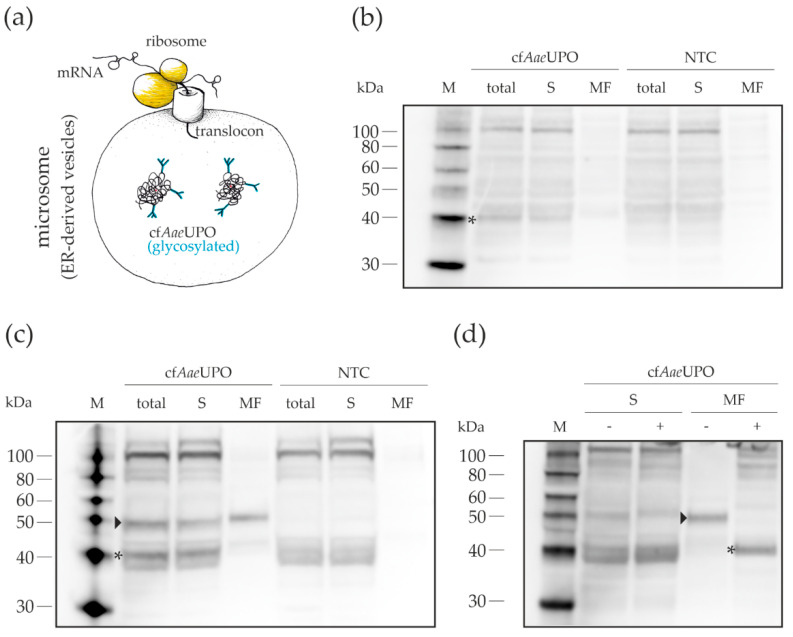
Localization of cell-free *Aae*UPO synthesized with lysates from *A. niger* and *N. crassa*, respectively. CFPS was done in 40-µL reactions, incubated at 18 °C for 5 h and fractionated; 10 µL of the whole CFPS reactions, supernatant, and microsomal fractions, respectively, were subjected to SDS-PAGE and subsequent Western blot analysis using a polyhistidine tag antibody. (**a**) Schematic representation of the cell-free synthesis of *Aae*UPO. (**b**) CFPS samples from *A. niger*. (**c**) CFPS samples from *N. crassa*. (**d**) Deglycosylation of cf*Aae*UPO produced with lysate from *N. crassa*. (−)—without deglycosylation enzyme mix; (+) with deglycosylation enzyme mix; M—protein marker, total—whole CFPS reaction; S—supernatant; MF—microsomal fraction; NTC—no-template control; arrowhead—glycosylated cf*Aae*UPO; asterisk—non-glycosylated cf*Aae*UPO.

**Figure A2:**
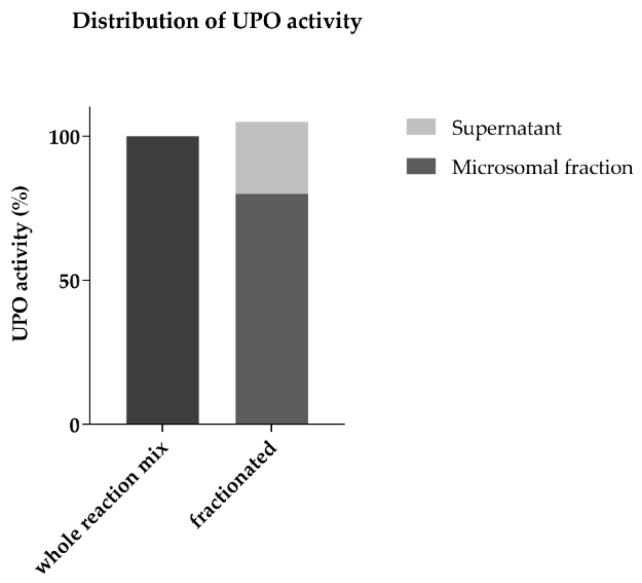
Distribution of UPO activity in the supernatant and the microsomal fraction. 20-µL CFPS reactions with *Aae*UPO mRNA were incubated at 18 °C for 4 h and separated in supernatant and microsomal fraction by centrifugation. The whole reaction mix and fractions were tested for UPO activity by measuring the conversion of propranolol to 5-OHP by LC-MS. Data given for the supernatant and microsomal fraction were calculated as percentages of activity in the whole reaction mixture.

Western blot analyses were done to get information about the localization of the synthesized protein and its relative amount (Figure 3). As a secretory protein, cf*Aae*UPO was expected to be present in the microsomes, i.e., in ER-derived vesicles. In the case of the *A. niger* lysate-based CFPS, a band was detected in the microsomal fraction that corresponded to *Aae*UPO, which has a theoretical molecular weight of 41.5 kDa in its unprocessed form. Overall, the amount of detectable protein was rather low, which explains the relatively low activity. With the *N. crassa* lysate, two prominent bands could be seen in the whole CFPS reaction mixture. As demonstrated by deglycosylation (Figure 3d), the lower band corresponds to the non-glycosylated (marked with an asterisk) and the upper band to the glycosylated (marked with an arrowhead) form of cf*Aae*UPO. The larger part of the glycosylated protein was located in the microsomes, while the non-glycosylated UPO was almost exclusively found in the supernatant. Activity measurements of the fractions indicated that the UPO activity stems from the glycosylated form as the ratio of the activities between the fractions correlated with the distribution of synthesized protein (Figure A2). The residual glycosylated cf*Aae*UPO seen in the supernatant fraction may be the result of an incomplete fractionation process, similarly described in an article by Thoring et al. [41]. They showed that—after centrifugation with 16,000× *g*—smaller microsomes were still present in the supernatant. The rather high portion of non-glycosylated protein might indicate an insufficient translocation capacity. Nonetheless, the successful expression of active *Aae*UPO proves that our fungal lysates contain functional microsomes that allow appropriate post-translational modification of secretory proteins. 

Unfortunately, it was not possible to use the polyhistidine tag on the cf*Aae*UPO for purification from the CFPS reaction with sufficient purity, due to the presence of a larger amount of contaminating fungal proteins. Thus, an accurate protein concentration of the synthesized cf*Aae*UPO could not be determined. In future experiments, the use of more appropriate affinity tags, such as the Strep II tag, should be considered [42].

**Figure 4:**
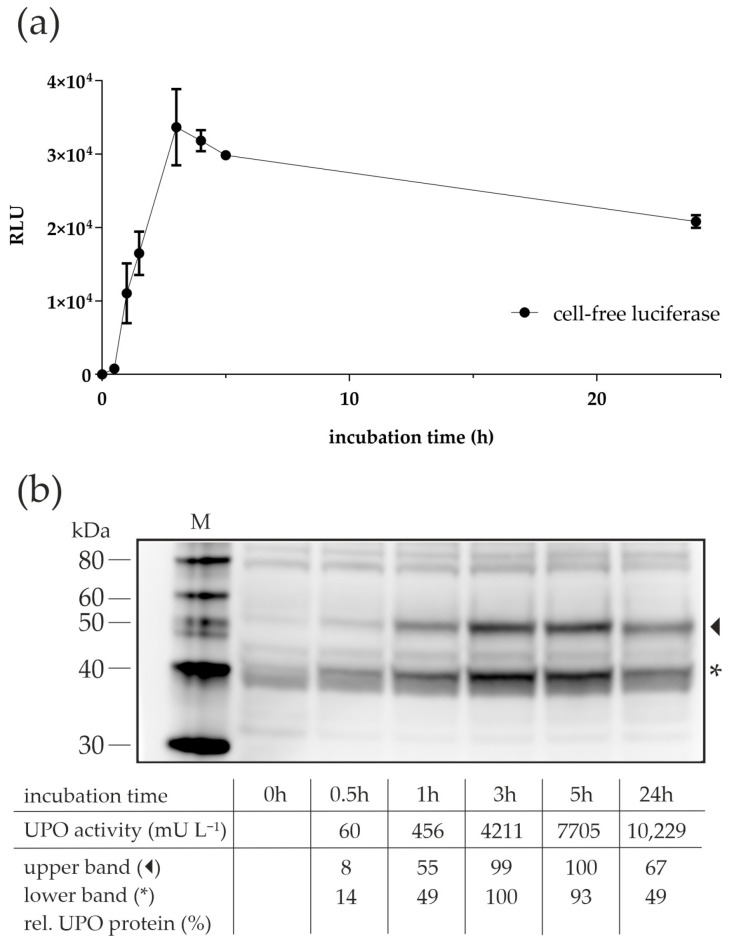
Time-dependent cell-free protein synthesis of luciferase and *Aae*UPO with *N. crassa* lysate. (**a**) Detection of luciferase activity in CFPS-batch reactions at different time points. The 20-µL reactions were prepared in separate tubes, incubated at 18 °C for the indicated time, and tested for luciferase activity by measuring the oxidation of luciferin via luminescence detection. Values are means ± standard deviation. (**b**) Western blot analysis of CFPS samples from different incubation times. The 20-µL reactions were prepared in separate tubes, incubated at 18 °C for the indicated time, and subjected to SDS-PAGE and, subsequently, Western blot analyses using a polyhistidine tag antibody. The semi-quantitative analyses were done individually for the glycosylated and non-glycosylated proteins. The densitometric values for t0 were subtracted from the values of other samples and the highest values were set to 100%. M—protein marker; arrowhead—glycosylated cf*Aae*UPO; asterisk—non-glycosylated cf*Aae*UPO.

The good results for the long incubation times with the lysate from *N. crassa* (Figure 2) were unexpected since, in batch CFPS reactions, the synthesis process usually stops after a few hours. For example, Hodgman and Jewett reported a “long synthesis time of 120 min” for luciferase using a yeast CFPS-batch reaction system, albeit at a slightly higher incubation temperature of 21 °C [37]. The main reasons for the termination of synthesis were seen in a rapid decline in energy-providing components and an accumulation of inhibitory molecules, such as inorganic phosphate [43]. To clarify whether the apparently long synthesis time in our experiments was actually due to better protein synthesis performance of the lysate, we expressed the reporter protein luciferase using CFPS with the lysate of *N. crassa* and measured the activity over a 24-hour period (Figure 4a). The activity of luciferase increased over the first three hours of CFPS but decreased thereafter, suggesting that the process of protein synthesis was not maintained throughout the incubation period. To confirm that this was also true for CFPS of *Aae*UPO, samples from the time-course experiment (Figure 2) were subjected to Western blot analysis (Figure 4b). As in the previous Western blot, two prominent bands appeared in the gel. Their intensity and thus the amount of cf*Aae*UPO produced increased until the third hour of incubation and then remained nearly constant (or decreased slightly) for the next hours. The 24-hour samples appeared to have lower cf*Aae*UPO protein levels than the samples from the previous time points, although activity was highest after 24 h. It might be that nonfunctional, e.g., misfolded UPO protein was degraded by remaining proteases in the lysate, but this needs to be clarified in future experiments.

Since the capacity to produce protein was limited to 3 h in our setup, an intrinsic maturation period of the UPO protein could be an explanation for the apparently long synthesis time. Such a maturation time was observed for a bacterial P450 monooxygenase that was expressed in a cell-free approach using lysate from *E. coli* [25]. In this case, the highest enzyme activity was reached 150 min after protein synthesis had stopped. However, since the exact process of heme incorporation and protein folding is still unknown for UPOs, it must be clarified in the future whether a longer maturation time is indeed necessary. Here, the CFPS might prove to be a valuable tool for gaining insight into the genesis of UPOs. It should be relatively simple to change the conditions in CFPS, for example, by adding mRNA for the co-expression of chaperones. The effects on UPO formation would be directly measurable and could be evaluated in a short time.

Table 1 gives a brief comparison of the volume activities and production times of *Aae*UPOs obtained from homologous, heterologous, or cell-free approaches. Attempts to express wt*Aae*UPO heterologously in the standard expression hosts *S. cerevisiae* or *P. pastoris* with appreciable yields failed [44,45]. Rounds of directed evolution were necessary to increase the production in the two yeasts that do not contain endogenous UPO genes [20,45]. This highlights an advantage of the cell-free approach: the possibility to use translationally active lysates from naturally UPO-coding (and producing) fungi. Thereby, all essential components should be available for the in vitro UPO synthesis. However, it has to be mentioned that the evolved mutant variant r*Aae*UPO PaDa-I expressed in *P. pastoris* in a bioreactor in a six-day cultivation period reached much higher expression levels than the homologous or cell-free systems. Nevertheless, cell-free protein production is by far the fastest method to gain significant amounts of functional UPO.

If the heterologously produced PaDa-I variant of r*Aae*UPO is included in the comparison of the three expression systems, the yield obtained with CFPS appears to be the lowest. However, in the present study, we have not yet fully exploited the potential of the cell-free approach using fungal lysates. There are general aspects in CFPS that have a high impact on the performance and would need to be optimized, for example, biomass production, lysate preparation, and buffer composition [46,47]. Also, the template design is essential for an optimal translation initiation, which can be triggered by adding elements like IRES [48]. As indicated by the Western blot analyses (Figure 3), the production of active UPO in our setup may be limited by a non-optimal translocation efficiency. Co-expressing components of the secretory apparatus could solve this problem, as this approach was already successful in living cells, for example, for the production of secreted antibodies in CHO cells [49]. Additionally, there is the possibility to change the CFPS reaction from a batch to a continuous process, which would result in an increase in protein yield [50]. Other influential parameters or components are specific for the expression of UPOs. The availability and fit of the heme cofactor are crucial for the functioning of UPOs, therefore the formulation and amount of heme in CFPS reactions will have to be optimized. This could be achieved on different levels: (i) during growth of the fungi, e.g., with the addition of hemin, which worked well in the expression of horseradish peroxidase in *P. pastoris* [51]; and (ii) by adding heme directly to the CFPS reaction, which was successful in the cell-free production of manganese peroxidase as well as horseradish peroxidase [23,24,27]. Since *Aae*UPO and other UPOs have at least one internal disulfide bond and other UPOs form functional dimers via disulfide bridges, it might also be beneficial to add protein disulfide isomerases like PDI or DsbC to CFPS reactions [23,27].

### 3.3. Substrate Screening with Cell-Free AaeUPO

**Figure A3:**
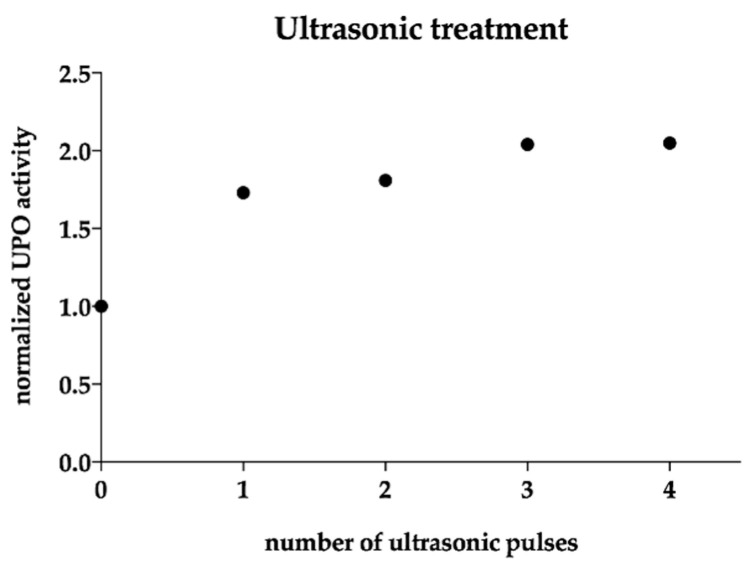
Release of cell-free produced peroxygenase (cf*Aae*UPO) from microsomes by sonication; a 320-µL CFPS reaction in a 1.5-mL reaction tube was sonicated with the indicated number of pulses (10 s, with 30 s pause interval). After each pulse, a 15-µL sample was taken. All samples were centrifuged, and the supernatants were tested for UPO activity by measuring the conversion of propranolol to 5-OHP by LC-MS. UPO activities were normalized in relation to the untreated sample.

The cell-free protein production approach should offer the possibility to establish a rapid screening platform for interesting biocatalysts. Thus, we tested the cell-free UPO produced with *N. crassa* lysate directly in a substrate screening without the need for time-consuming purification steps. To this end, the conversion (oxygenation, oxidation) of typical UPO substrates, which are also used in activity assays, as well as of three common pharmaceuticals was performed with simply prepared CFPS reaction solutions. The enzyme that is partly located in microsomes was efficiently released by ultrasonic treatment (Figure A3). After centrifugation, the supernatant was used for photometric UPO assays with ABTS, veratryl alcohol, 5-nitrobenzodioxole, and naphthalene, and for LC-MS analyses with the pharmaceuticals propranolol, diclofenac, and clopidogrel.

**Figure 5:**
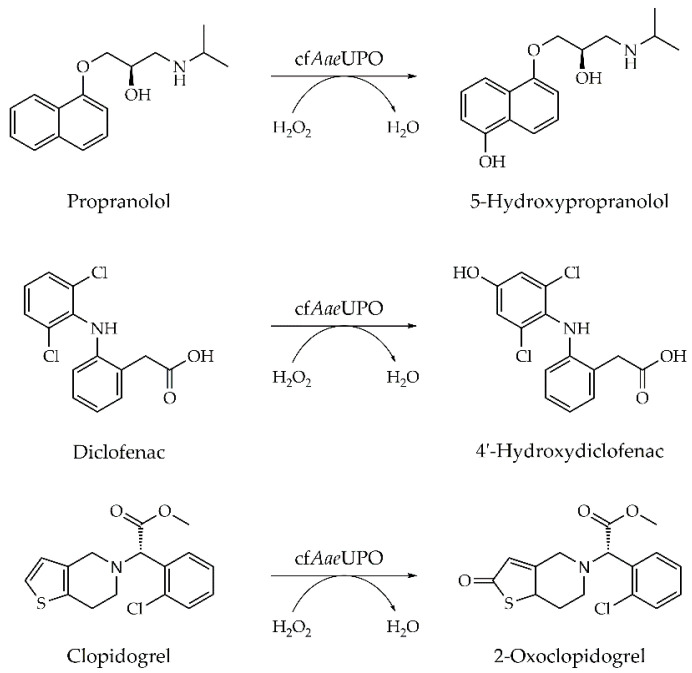
Conversion of the pharmaceuticals propranolol, diclofenac, and clopidogrel by cell-free prepared UPO (cf*Aae*UPO).

The β-adrenergic blocker propranolol, the non-steroidal anti-inflammatory drug diclofenac, and the antithrombotic prodrug clopidogrel are known to be metabolized by cytochrome P450 monooxygenases in the human body. In previous studies, it was shown that wt*Aae*UPO is able to perform the same reactions to give the (major) human drug metabolites 5-hydroxypropranolol, 4′-hydroxydiclofenac, and 2-oxoclopidogrel, respectively (Figure 5) [15,40,52].

The activities of cf*Aae*UPO were compared with those of its wild-type counterpart (taking into account that wt*Aae*UPO did not contain a polyhistidine tag). For the purpose of comparison, we used enzyme samples with a normalized volume activity of 100 U L^−1^ (based on veratryl alcohol oxidation) in all reactions. The results are listed in Table 2.

All substrates were converted by both enzyme preparations, although quantitative differences in the activities were found in some cases. For NBD, naphthalene, and propranolol, the measured activities were similar; however, for ABTS, diclofenac, and clopidogrel, the activities of the cell-free variant were two to four times lower. ABTS is a substrate that is oxidized via one-electron abstraction by the peroxidative activity of UPO resulting in a cation radical product (‘classic’ peroxidase activity without oxygen transfer). The fungal lysate used for CFPS may have contained metabolites with antioxidant properties that could have interfered with the detection of the ABTS radical [53]. Thus, substrates like ABTS might only be suitable in screening approaches with unpurified UPO, if the yield of cell-free enzyme is relatively high. A possible explanation for the lower conversion of diclofenac and clopidogrel could be the presence of the C-terminal polyhistidine tag, which may have impaired activity, e.g., by shielding the heme-access channel. Such an effect was already reported for a mutant variant of *Aae*UPO (PaDa-I) [54]. In addition, negative effects of potentially different glycosylation patterns cannot be excluded.

In summary, it took less than two days from the DNA template carrying the *Aae*UPO gene to obtain the substrate screening results with expressed cf*Aae*UPO. This is much faster compared to conventional screening systems based on heterologous expression in host microbes [55]. Moreover, if transcription and translation were coupled in one CFPS reaction, the turnaround time of the entire process could be reduced even further.

## 4. Conclusions

In the present study, we evaluated a novel approach to produce unspecific peroxygenases (UPOs) by using a cell-free synthesis system based on fungal lysates. The UPO from *Cyclocybe (Agrocybe) aegerita* was successfully expressed with lysates derived from *N. crassa* or *A. niger*. Activities of this cf*Aae*UPO of up to 105 U L^−1^ (based on veratryl alcohol oxidation) could be achieved within 24 h, where the lysate from *N. crassa* showed a significantly higher translational performance compared to *A. niger*. The unpurified CFPS reaction solutions were subjected to a substrate screening, in which the performance of the cell-free enzyme (cf*Aae*UPO) was comparable to that of the homologously produced enzyme (wt*Aae*UPO). The procedure presented here highlights the general potential of CFPS for rapid screening of new UPOs or UPO variants, including the estimation of their catalytic performance. Nevertheless, for large-scale production of suitable candidates, hetero-logous production in fungal hosts will continue to be the method of choice, as cell-free synthesis is currently not competitive in terms of yield. Last, but not least, it must be pointed out that the full potential of lysates from filamentous fungi is far from being tapped, as their use in CFPS is still in its infancy.

## Figures and Tables

**Table 1:** Comparison of *Aae*UPO produced by different approaches.

Origin of *Aae*UPO	Homologous Expression (wt*Aae*UPO)	Heterologous Expression ^1^ (r*Aae*UPO)	Cell-Free Expression (cf*Aae*UPO)
Activity (veratryl alcohol U L^−1^)	1550	<1	105
Production time	10 days	6 days	24 h
Reference	[17]	[44]	present study

^1^ Further information is given in the text.

**Table 2:** Substrate conversion by homologous wt*Aae*UPO and cf*Aae*UPO. All reactions contained a UPO activity of 100 mU mL^−1^ (based on the oxidation of veratryl alcohol). The enzyme activities are given in mU mL^−1 (a)^ or µU mL^−1 (b)^.

	wt*Aae*UPO	cf*Aae*UPO
ABTS ^(a)^	414 ± 35	229 ± 24
5-Nitrobenzodioxole ^(a)^	90 ± 12	93 ± 8
Naphthalene ^(a)^	209 ± 14	212 ± 22
Propranolol ^(b)^	7505 ± 103	9972 ± 69
Diclofenac ^(b)^	10,701 ± 73	4875 ± 134
Clopidogrel ^(b)^	8949 ± 496	2616 ± 86

## Data Availability

The data presented in this study are available on request from the corresponding author.
